# Development and validation of an early warning model for postoperative reflux in patients with esophageal cancer

**DOI:** 10.1016/j.apjon.2025.100764

**Published:** 2025-07-25

**Authors:** Xueying Sun, Lirong Wei, Yuqing Zhao, Qian Bai, Na Cao, Sihan Liu, Huixia Li

**Affiliations:** Esophageal Oncology Department, Tianjin Medical University Cancer Institute & Hospital, National Clinical Research Center for Cancer, Tianjin's Clinical Research Center for Cancer, Key Laboratory of Cancer Prevention and Therapy, Tianjin, China

**Keywords:** Esophageal cancer, Postoperative reflux, Risk prediction, Early warning model, Logistic regression

## Abstract

**Objective:**

To develop and validate a predictive model for assessing the risk of postoperative reflux in patients with esophageal cancer.

**Methods:**

A total of 278 postoperative patients with esophageal cancer admitted between January and December 2022 were enrolled as the modeling cohort. An independent cohort of 120 patients admitted between January and April 2023 was used for external validation. Logistic regression analysis was performed to construct the risk prediction model. Model performance was evaluated in terms of discrimination, calibration, and goodness-of-fit. Discrimination was assessed using the area under the receiver operating characteristic curve (AUC), calibration was examined using calibration plots generated by 1000 bootstrap resamples, and the Hosmer–Lemeshow goodness-of-fit test was applied (*P* ​< ​0.05 considered significant).

**Results:**

The incidence of postoperative reflux in the modeling cohort was 36.3%. The AUC was 0.813 (95% CI: 0.762–0.864) in the modeling cohort and 0.820 (95% CI: 0.747–0.894) in the validation cohort, indicating good discriminative ability. The Hosmer–Lemeshow test showed *P*-values of 0.155 and 0.059 in the modeling and validation cohorts, respectively, indicating acceptable goodness-of-fit. Calibration curves demonstrated close agreement between predicted and observed probabilities in both cohorts, confirming good model calibration.

**Conclusions:**

The developed early warning model demonstrates acceptable predictive performance for identifying postoperative reflux risk in patients with esophageal cancer. It may assist clinicians in early risk assessment and individualized intervention, although further external validation is warranted prior to clinical implementation.

## Introduction

Esophageal cancer, one of the most prevalent malignant tumors of the digestive system stands as a more and more significant health concern worldwide. According to the latest data from the International Agency for Research on Cancer of the World Health Organization (IARC), the number of new cases and deaths of esophageal cancer in China is 604,000 and 544,000 respectively,^1^ highlighting its devastating impact on public health and mortality rates.^2^ Studies have also shown that the 5-year survival rate of esophageal cancer is approximately 15–25%, making it the most lethal cancers in the world.^3^ Meanwhile, the treatment of esophageal cancer advocates a comprehensive treatment based on surgical resection.^4^ However, gastro-esophageal reflux (GERD) is a common complication after esophageal cancer surgery due to the reconstruction of the digestive tract, which changes the normal anatomical position of stomach and causes the disruption of the anti-GERD mechanism.^5^ GERD refers to a dysfunction in which gastric contents, including bile salts and pancreatic enzymes that flow from the duodenum into the stomach, reflux into the esophagus.^6^ Studies have shown^7^ that postoperative patients with esophageal cancer are particular susceptible to GERD reflux. This common complication after esophageal cancer surgery severely affects the quality of patient survival.^8^ Due to the stimulation of reflux, patients often suffer from acid reflux, irritating cough, sore throat, heartburn, and other uncomfortable symptoms after eating.^9^ Long-term exposure to reflux can lead to organic damages, such as esophageal ulcers, esophageal bleeding, and other related conditions. In severe cases, choking and lung infection may also occur due to the accidental inhalation of reflux and ultimately lead to death.^10^ Therefore, improving gastroesophageal reflux symptoms in postoperative patients with esophageal cancer is an important indicator to improve the quality of patients' postoperative survival. Early warning and prevention of reflux in postoperative patients with esophageal cancer are pivotal in managing this condition. While considerable research has focused on symptomatic nursing interventions for postoperative patients with esophageal cancer,^11^, ^12^, ^13^ there is still a notable scarcity of studies on exploring effective strategies to predict and detect reflux of patients at an early stage, as well as to prevent and alleviate its occurrence. Timely and accurate interventions are essential for improving postoperative outcomes and prognosis in patients with esophageal cancer with reflux. The development of such a model aims to identify risk factors associated with postoperative reflux, thereby equipping clinical nursing staff with a tool for early detection and timely intervention. This, in turn, could lead to improved management of reflux symptoms and enhanced quality of life for postoperative patients with esophageal cancer. Hence, the present study endeavors to construct an early warning model for postoperative reflux in patients with esophageal cancer, exploring its potential to guide clinical practice and inform early interventions.

## Methods

### Subjects

This retrospective study employed the convenience sampling method to analyze the patients who were hospitalized in a tertiary-level A-class oncology hospital in Tianjin from January 2022 to April 2023 and underwent radical esophageal cancer surgery and met the inclusion and exclusion criteria. This study aimed to standardize surgical treatment and perioperative nursing procedures to ensure consistency in clinical practice. The modeling group and validation group were divided using a time-split method. Specifically, all eligible patients with esophageal cancer who underwent surgery and were enrolled consecutively from January 2022 to December 2022 (*n* ​= ​278) were assigned to the modeling group; subsequent patients enrolled consecutively from January 2023 to April 2023 (*n* ​= ​120) were assigned to the validation group. Both groups underwent identical surgical procedures performed by the same surgical team, standardized tubular gastric reconstruction techniques, and consistent postoperative care protocols. The time-split design guaranteed that validation group data were independent of model development.

Inclusion criteria: (1) Patients diagnosed with esophageal cancer by histology or pathology; (2) Patients who underwent surgery for esophageal cancer and underwent reconstruction of the digestive tract with a tube stomach; (3) Patients who entered the dietary transition period within one week post-surgery; (4) Patients aged 18 years old or older; (5) Patients who provided informed consent for study participation; (6) Patients being able to complete the questionnaire by themselves or with the assistance of their carers. Exclusion criteria: (1) patients with esophageal hiatal hernia, or previous history of pharyngolaryngeal or gastroesophageal surgery; (2) patients with serious postoperative complications such as anastomotic fistula that require fasting; (3) patients with cognitive dysfunction. Removal criteria: (1) death due to serious complications during the study period; (2) invalid or incomplete data.

**Fig. 1:**
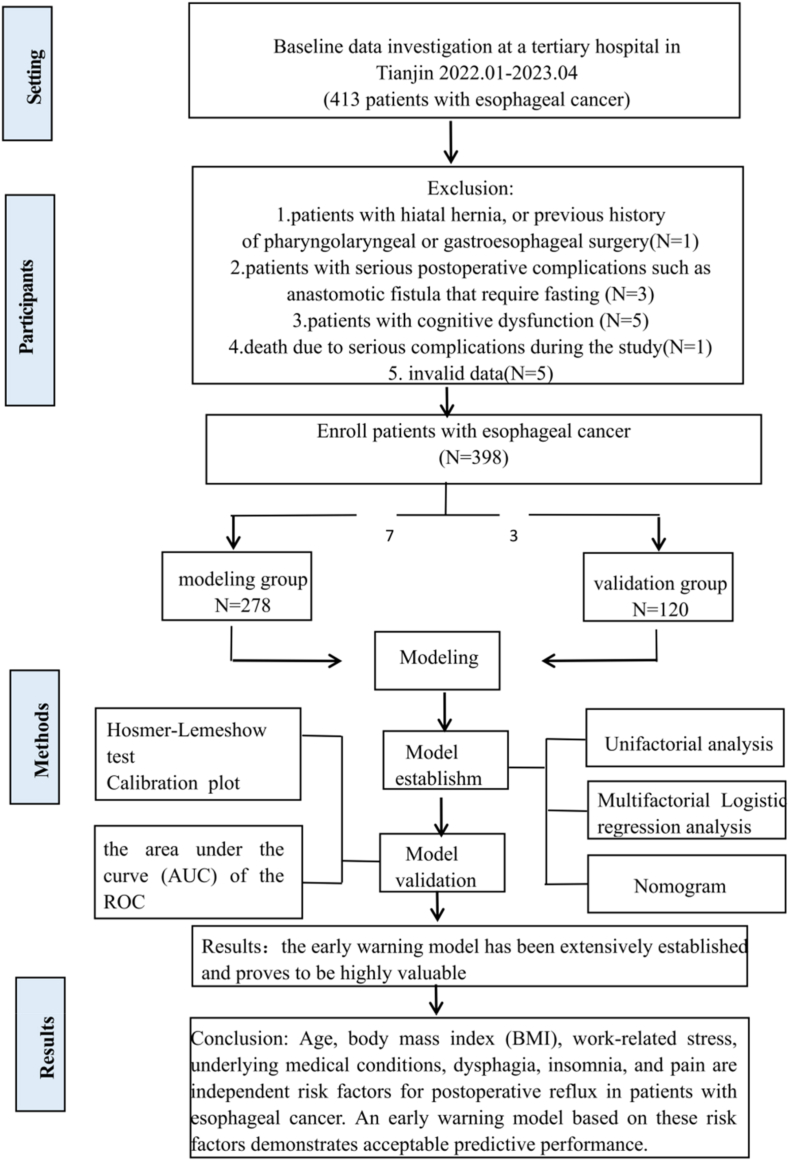
Study design flow chart.

A total of 29 risk factor variables that may be related to reflux in postoperative patients with esophageal cancer were included in this study. Sample size estimation was based on the principle of impact factor studies, suggesting a ratio of 5–10 times the number of variables.^14^ In this study, 10 times the number of variables was taken, i.e., 290 cases; and considering that there was information on ineligible cases, the sample continued to be enlarged by 15%, i.e., the sample size was determined to be at least 341 cases. Based on the sample size requirements for reference prediction models^15^ (the recommended ratio of 7:3 between the modeling and verification groups), this study ultimately included 413 patients, Excluded patients included those patients with hiatal hernia, or previous historyof pharyngolaryngeal or gastroesophageal surgery (*n* ​= ​1); patients with serious postoperative complications such as anastomotic fistula that require fasting (*n* ​= ​3); patients with cognitive dysfunction (*n* ​= ​5); death due to serious complications during the study (*n* ​= ​1); In addition, regarding missing data, since there was little missing data in this study, only five cases of invalid missing data were identified, and these data had a negligible impact on the overall data analysis. Therefore, these data were deleted (*n* ​= ​5). A total of 398 patients completed the study. Among these, 278 patients were assigned to the modeling group and 120 to the validation group. Through follow-up (in-hospital follow-up, telephone follow-up, and outpatient follow-up), patients who developed reflux symptoms were identified, and the modeling group was further divided into a reflux group and a non-reflux group. Fig. 1 shows the flow chart of the study design.

### Research tools

#### General information questionnaire

The risk factors incorporated in this study were identified through a systematic review of existing literature,^16^, ^17^, ^18^, ^19^ supplemented by a standardized consultative process involving a panel of 10 senior experts specializing in esophageal tumors, oncology nursing, and epidemiology. There is no denying for the potential existence of certain unaccounted-for factors, including genetic predisposition, postoperative medication differences, etc. However, this study primarily focuses on modifiable factors such as demographic characteristics, lifestyle, and postoperative symptoms. Future endeavors encompass broadening the range of variables through multicenter collaboration and optimizing variable selection, while comprehensively considering the availability and practicality of clinical data. Ultimately, the potential risk factors for reflux will be established, include (1) general condition: age, gender, Body Mass Index (BMI), work stress; (2) lifestyle behaviors: whether consuming strong tea, high salt diet, spicy diet, overeating, lying down after eating, smoking, alcohol consumption, maintaining bed height during sleep, (3) disease-related factors: underlying disease, tumor location, whether neoadjuvant therapy was administered before surgery, and the occurrence of related symptoms (including dysphagia, fatigue, insomnia, pain, diarrhea, constipation, cough, nausea and vomiting, etc.).

#### Gastroesophageal reflux disease questionnaire (GerdQ)

The GerdQ questionnaire, developed by Dent et al., in 2007, serves as a standardized tool for the diagnosis and assessment of gastroesophageal reflux disease (GERD). It comprises six items: four positive questions addressing typical reflux symptoms and two negative questions evaluating symptoms unrelated to reflux. Participants are asked to recall the frequency of each symptom over the past seven days. Responses are graded on a four-point scale. For positive items, scores increase with symptom frequency: 0 (no symptoms), 1 (1 day), 2 (2–3 days), and 3 (4–7 days). In contrast, for negative items, scores decrease with increasing symptom frequency: 3 (no symptoms), 2 (1 day), 1 (2–3 days), and 0 (4–7 days). The total GerdQ score is calculated by summing all six items, yielding a range from 0 to 18. Higher scores indicate a greater likelihood of GERD.^20^^,^^21^

Several studies have demonstrated that the GerdQ has strong clinical utility in diagnosing gastroesophageal reflux disease (GERD).^22^, ^23^, ^24^ It has been validated as a concise, rapid, noninvasive, cost-effective, and safe diagnostic instrument. In addition to objectively assessing reflux-related symptoms, the GerdQ also captures the disease's impact on patients' quality of life^20^^,^^21^ and other related clinical outcomes.^25^^,^^26^ According to the GerdQ Research Group and related studies,^26^^,^^27^ a total score of 10 or higher is indicative of the presence of reflux. In the present study, the GerdQ was used to assess and determine the occurrence of postoperative reflux in patients with esophageal cancer.

### Data collection and quality assurance protocols

In the pursuit of rigorous data collection, adherence to predefined inclusion and exclusion criteria is paramount, accompanied by a transparent and meticulous case selection screening process. Trained researchers are expected to provide a detailed explanation of the purpose, methodologies, components, and precautions of the survey questionnaire to participants, aiming to foster active cooperation. Participants are instructed to fill out the questionnaire independently, and researchers are responsible for clarifying any unclear or disputed items in the questionnaire without any leading explanations. For participants with limited educational attainment or writing difficulties, researchers may engage in thorough communication with them to assist in completing the questionnaire objectively and accurately. For patients with poor memory, detailed instructions should be provided at the beginning of the questionnaire, explicitly urging participants to focus and recall relevant information from the past seven days. Moreover, to ensure comprehension and memory retrieval, simple and straightforward sentences rather than complex or ambiguous terminology are preferable to formulate questions to help respondents better understand and recall pertinent events. Prompt organization and analysis of the collected data, dual-person system for data coding, entry, and cross-verification are imperative to ensure data completeness and accuracy. Discrepancies should be resolved through consultation.

### Statistical analysis

Statistical processing in this study was facilitated by SPSS version 17.0 software, with R version 4.3.3 utilized for constructing column-line graphical models. Count data were expressed as cases and percentages, and the χ^2^ test was used for intergroup comparisons; non-normally distributed measures were expressed as medians and quartiles, with non-parametric tests used for intergroup comparisons. Binary logistic regression analysis was used to construct the early warning model. The discriminative ability of the model was evaluated using the area under the ROC curve (AUC), Calibration was evaluated using calibration curves and the Hosmer–Lemeshow (HL) test with the “rms” package. Differences were considered statistically significant at *P* ​< ​0.05. Candidate variables were initially screened based on the results of chi-square test or nonparametric test analysis (*P ​<* ​0.05) to avoid omission of potential confounders; variance inflation factor (VIF) was calculated, with VIF < 2.0 serving as the criterion for all included variables to mitigate the interference of multicollinearity.

## Results

### Incidence of postoperative reflux in patients with esophageal cancer

In the modeling group of this study, a total of 278 postoperative patients with esophageal cancer were amassed, with 101 experiencing reflux, yielding an incidence rate of 36.3%. In the validation group, a total of 120 postoperative patients with esophageal cancer were collected, of which 45 patients experienced reflux, with an incidence rate of 37.5%.

### Characteristics of patients with esophageal cancer who develop reflux after surgery

The results of the chi-square test and nonparametric tests revealed that there were statistically significant differences in age, BMI, work stress, presence of underlying diseases, preoperative treatment, occurrence of dysphagia, occurrence of insomnia, and occurrence of pain (*P* ​< ​0.05), as detailed in Table 1.

**Table 1:** Characteristics of patients with esophageal cancer who develop reflux post-surgery (*N* ​= ​278).

Item		Reflux *n* (%) (*n* ​= ​10)	No reflux *n* (%) (*n* ​= ​177)	Statistic (*Z/χ*^*2*^)	*P*-values∗
**Sex**	Male	81 (80%)	145 (82%)	0.04^a^	0.8
	Female	20 (20%)	32 (18%)		
**Age (years)**		62 (57, 65)	60 (54, 63)	10,430.5^b^	0.020
**BMI (kg/m^2^)**		23.12 (21.22, 25.18)	21.32 (18.49, 23.63)	12,491.5^b^	< 0.001
**Work pressure**	Yes	73 (72%)	83 (47%)	15.81^a^	< 0.001
	No	28 (28%)	94 (53%)		
**Drinking strong tea**	Yes	59 (58%)	113 (64%)	0.59^a^	0.4
	No	42 (42%)	64 (36%)		
**High salt diet**	Yes	52 (51%)	110 (62%)	2.58^a^	0.11
	No	49 (49%)	67 (38%)		
**Spicy diet**	Yes	58 (57%)	121 (68%)	2.89^a^	0.089
	No	43 (43%)	56 (32%)		
**Overeating**	Yes	64 (63%)	121 (68%)	0.51^a^	0.5
	No	37 (37%)	56 (32%)		
**Lying down or not**	Yes	63 (62%)	111 (63%)	< 0.001^a^	> 0.9
	No	38 (38%)	66 (37%)		
**Smoking**	Yes	70 (69%)	101 (57%)	3.57^a^	0.059
	No	31 (31%)	76 (43%)		
**Alcohol consumption**	Yes	51 (50%)	111 (63%)	3.46^a^	0.063
	No	50 (50%)	66 (37%)		
**Head height**	Yes	51 (50%)	94 (53%)	0.09^a^	0.8
	No	50 (50%)	83 (47%)		
**Presence of underlying disease**	Yes	35 (35%)	92 (52%)	7.1^a^	0.008
	No	66 (65%)	85 (48%)		
**Tumor location**	Middle	53 (52%)	98 (55%)	0.12^a^	0.7
	Lower segment	48 (48%)	79 (45%)		
**Preoperative treatment**	No	64 (63%)	86 (49%)	6.31^a^	0.043
	Chemotherapy	31 (31%)	70 (40%)		
	Chemotherapy ​+ ​radiotherapy	6 (6%)	21 (12%)		
**Swallowing difficulty**	Yes	75 (74%)	98 (55%)	8.98^a^	0.003
	No	26 (26%)	79 (45%)		
**Fatigue occurs**	Yes	48 (48%)	106 (60%)	3.49^a^	0.062
	No	53 (52%)	71 (40%)		
**Insomnia**	Yes	46 (46%)	106 (60%)	4.77^a^	0.029
	No	55 (54%)	71 (40%)		
**Occurrence of pain**	Yes	75 (74%)	91 (51%)	13.02^a^	< 0.001
	No	26 (26%)	86 (49%)		
**Occurrence of diarrhoea**	Yes	45 (45%)	92 (52%)	1.14^a^	0.3
	No	56 (55%)	85 (48%)		
**Constipation**	Yes	59 (58%)	99 (56%)	0.08^a^	0.8
	No	42 (42%)	78 (44%)		
**Coughing**	Yes	68 (67%)	99 (56%)	3.02^a^	0.082
	No	33 (33%)	78 (44%)		
**Nausea vomiting**	Yes	66 (65%)	128 (72%)	1.17^a^	0.3
	No	35 (35%)	49 (28%)		

BMI, body mass index.

∗ Nonparametric tests; chi-square test.

a *χ*^*2*^ value.

b *Z* value.

### Multifactorial logistic regression analysis of factors influencing the occurrence of postoperative reflux in EsophagealCancer patients

This study first conducted a preliminary screening of 29 potential variables using chi-square tests or nonparametric tests. Eight variables were found to be significantly associated with the risk of reflux occurrence at a significance level of *P* ​< ​0.05, and were then included in a logistic regression model, as shown in Table 2. The model was further optimized using stepwise regression (removing variables with *P* ​≥ ​0.05).

**Table 2:** The way of assigning values to the independent variables.

Independent variables	Assignment method
Age	Entered as the original value
BMI	Input as the original value
Working pressure	Yes ​= ​1; No ​= ​0
Presence of underlying disease	Yes ​= ​1; No ​= ​0
Pre-operative treatment	Chemotherapy ​+ ​radiotherapy ​= ​3; Chemotherapy ​= ​2; No ​= ​1
Dysphagia	Yes ​= ​1; No ​= ​0
Insomnia	Yes ​= ​1; No ​= ​0
Occurrence of pain	Yes ​= ​1; No ​= ​0

BMI, body mass index.

**Fig. 2:**
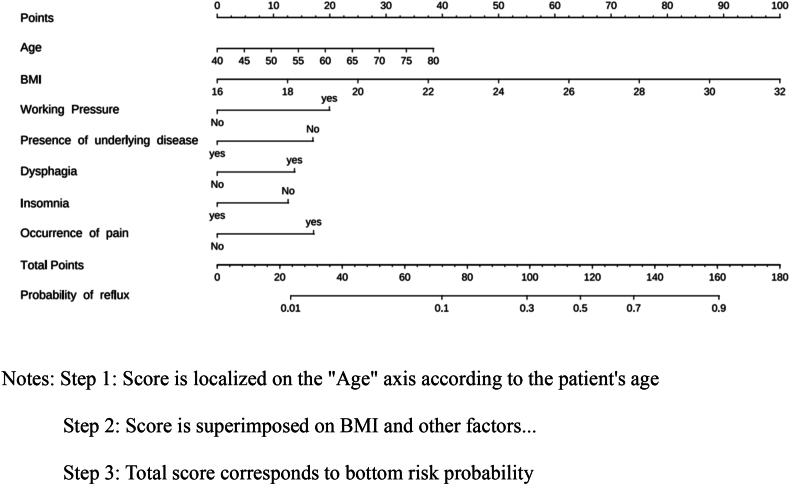
Early warning model for the occurrence of reflux after surgery in patients with esophageal cancer.

**Fig. 3:**
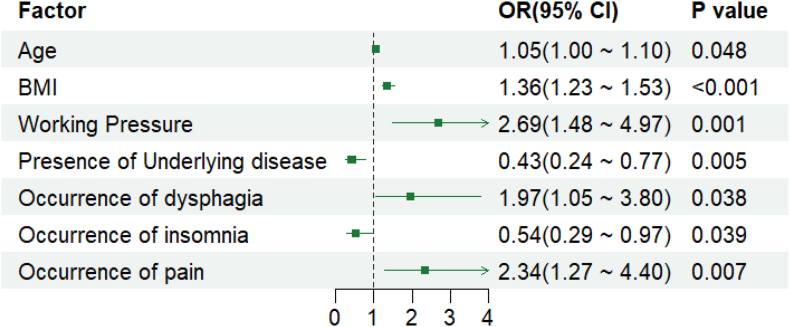
Forest plot of predictor odds ratios for the occurrence of reflux after surgery in patients with esophageal cancer.

Notably, preoperative treatment variables did not meet the significance threshold and were excluded from the final model, thereby resulting in a final model retaining 7 statistically significant predictive variables: age, BMI, work stress, presence of underlying conditions, occurrence of dysphagia, insomnia, and pain. These variables were identified as influencing factors for occurrence of reflux in patients with esophageal cancer after surgery, with specific results shown in Table 3. The final risk prediction model for postoperative reflux in patients with esophageal cancer was formulated as follows: Logit(*P*) ​= ​0.048 ​× ​age ​+ ​0.310 ​× ​BMI ​+ ​0.988 ​× ​work-related stress ​− ​0.842 ​× ​presence of underlying conditions ​+ ​0.681 ​× ​occurrence of dysphagia − 0.624 ​× ​occurrence of insomnia + 0.849 ​× ​occurrence of pain. The nomogram is shown in Fig. 2. And plotted forest plots of logistic regression coefficients to increase the visualization of regression coefficients, as shown in Fig. 3.

**Table 3:** Multifactorial Logistic regression analysis of factors influencing the occurrence of reflux in patients with esophageal cancer after surgery.

Factor	*β* value	*SE*	Wald *χ*^*2*^ value	*P* value	*OR* value	95% *CI*
Constant	−11.156	2.082	−5.358	< 0.001	0.00	0.00, 0.00
Age	0.048	0.024	1.973	0.048	1.05	1.00, 1.10
BMI	0.310	0.056	5.517	< 0.001	1.36	1.23, 1.53
Working pressure	0.988	0.308	3.208	0.001	2.69	1.48, 4.97
Presence of underlying disease	−0.842	0.300	−2.806	0.005	0.43	0.24, 0.77
Occurrence of dysphagia	0.681	0.327	2.079	0.038	1.97	1.05, 3.80
Occurrence of insomnia	−0.624	0.303	−2.064	0.039	0.54	0.29, 0.97
Occurrence of pain	0.849	0.316	2.687	0.007	2.34	1.27, 4.40

BMI, body mass index; SE, standard error; OR, odds ratio; CI, confidence interval.

### Analysis of the fitting and predictive effect of the early warning model for postoperative reflux in patients with esophageal cancer

The modeling group and validation group were evaluated using the receiver operating characteristic (ROC) curve, Hosmer–Lemeshow test, and calibration curves to assess the predictive performance of the reflux probability prediction model, with the validation group's data used for internal validation.

**Fig. 4:**
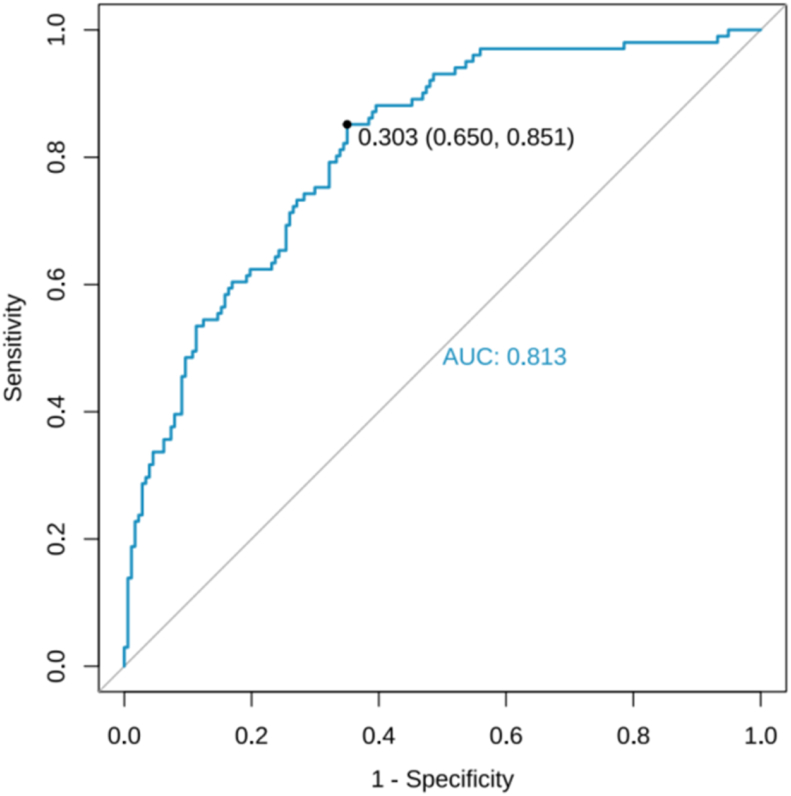
ROC curve of the Predictive Model in the modeling group. ROC, receiver operating characteristic.

**Fig. 5:**
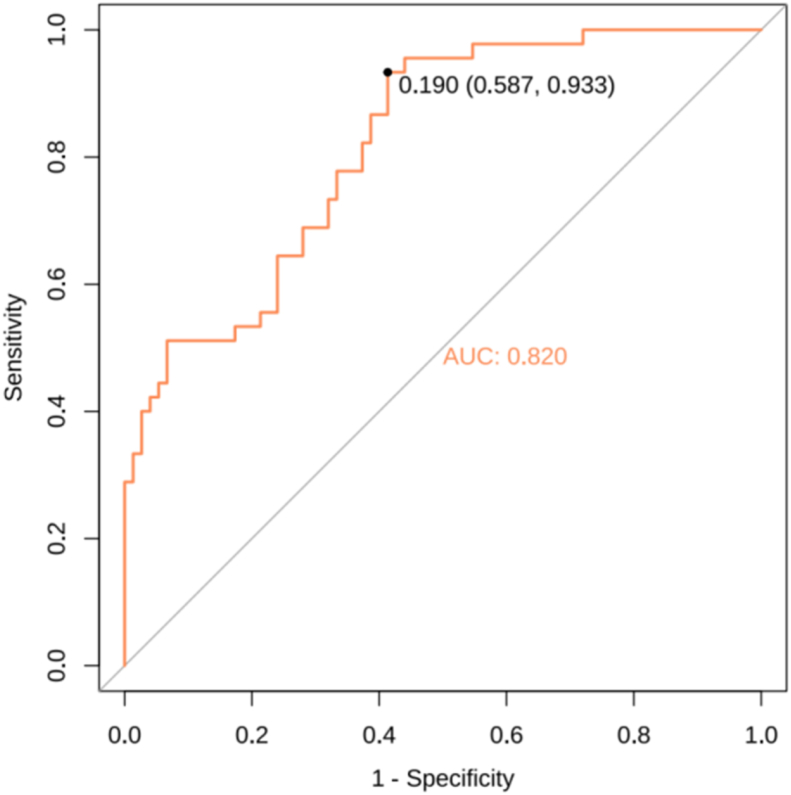
ROC curve of the Predictive Model in the validation group. ROC, receiver operating characteristic.

ROC curves were plotted for both the modeling group and validation group. A larger area under the ROC curve (AUC) indicates better discriminative ability of the predictive model. In this study, the AUC results for the modeling group were 0.813 (95% *CI*: 0.762–0.864), and the AUC results for the validation group were 0.820 (95% *CI*: 0.747–0.894), as presented in Fig. 4, Fig. 5.

**Fig. 6:**
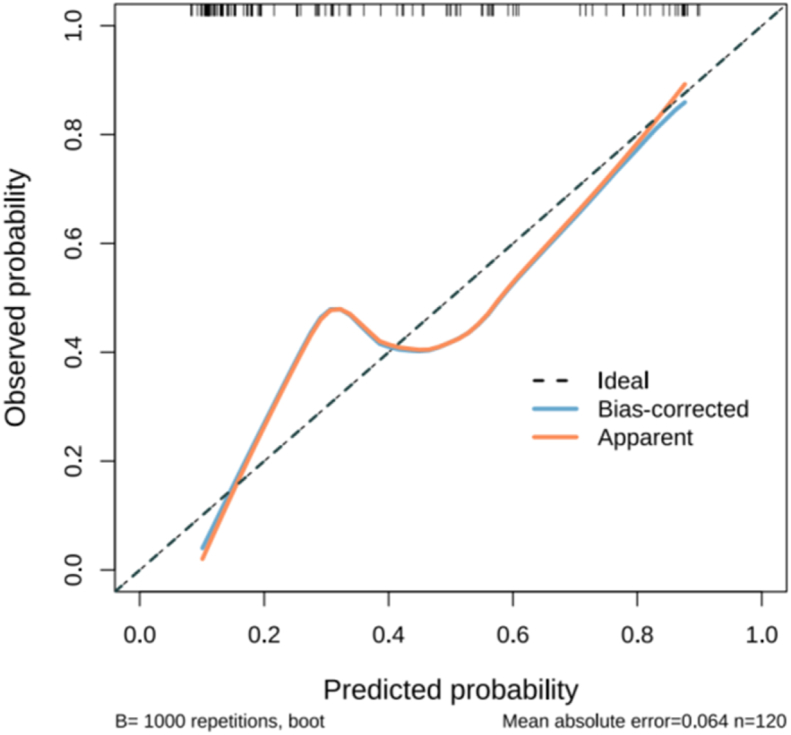
Calibration curve of the Predictive Model in the modeling group.

**Fig. 7:**
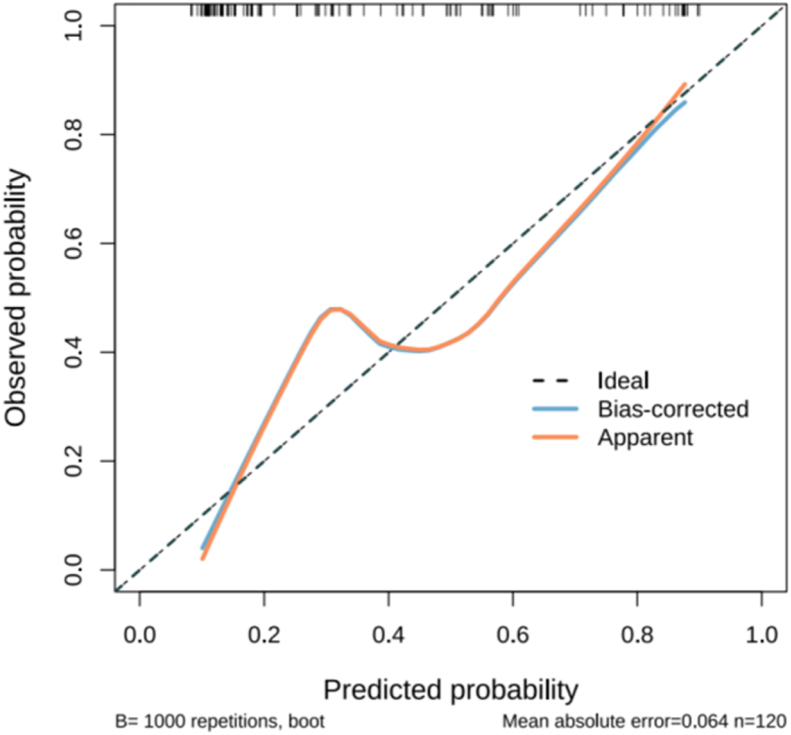
Calibration curve of the Predictive Model in the validation group.

The Hosmer–Lemeshow test results for the modeling group yield a *P*-value of 0.155, while those for the validation group yield a *P*-value of 0.059, indicating that the model has acceptable goodness-of-fit. The calibration curves obtained by bootstrapping 1000 times using the Bootstrap method highlight that the calibration curves of the modeling group prediction model (Fig. 6) and the validation group prediction model (Fig. 7) are generally close to the standard curves, Indicates the calibrated acceptable performance of the model.

## Discussion

### Higher incidence of postoperative reflux in patients with esophageal cancer

In this study, we investigated the incidence of postoperative reflux in patients with esophageal cancer, in which the incidence rate was 36.3% in the modeling group and 37.5% in the validation group. These findings were in conformity with the related studies, reinforcing the notion that patients with esophageal cancer face a heightened risk of postoperative reflux.^11^

The underlying causes of this phenomenon are multifaceted. Surgical procedures could destroy the integrity and physiological anatomical structure of the connection between the stomach and the esophagus, thereby resulting in abnormal anti-reflux function of the body; in addition, the peristaltic dysfunction of the residual esophagus after oesophagogastric partial resection also aggravates gastro-oesophageal reflux;^7^, ^28^ concurrently, the gastric tube exposed to the negative-pressure thoracic cavity is prone to dilation; and the pressure differences between the thorax and the abdominal cavity leads to remarkable increase in the patient's risk of gastro-oesophageal reflux during the postoperative period.^29^ Therefore, regarding the combination of multiple causes, patients with esophageal cancer are susceptible to gastro-oesophageal reflux after surgery. Symptoms such as sore throat, heartburn, and retrosternal burning pain induced by reflux^30^ and organic damage produced by long-term stimulation of reflux have adversely impacted the prognosis and quality of life of patients. Hence, alleviating the symptoms of gastroesophageal reflux in postoperative patients with esophageal cancer is crucial for enhancing their postoperative quality of life; and identifying how to better mitigate reflux frequency and ameliorate reflux symptoms in patients is of paramount importance. Consequently, early warning and preventive measures for reflux in postoperative patients with esophageal cancer are indispensable.

### Analysis of risk factors associated with postoperative reflux in patients with esophageal cancer

The results of this study underscored that age is a risk factor for the occurrence of reflux after surgery in patients with esophageal cancer, a 1-unit increase in age increases the risk of reflux by 5% (*OR* ​= ​1.05), with an elevated risk observed in older individuals, aligning with previous studies.^31^ This correlation can be attributed to aged-related degenerative modifications of multiple systems in the body, including gradual degeneration of esophageal sensory neurons. Conditions such as ineffective esophageal clearance, and luminal stagnation in the case of esophageal motility disorders can lead to an increased risk of reflux. Meanwhile, the heightened stress in elderly patients with esophageal cancer after surgery exacerbates the risk of postoperative stress-induced reflux; hence, for the esophageal cancer surgical patients in advanced age, the nurses should pay close attention, such as preoperative preaching to patients and their families, keeping the head of the bed elevated by more than 30° in postoperative daily recumbency, and advocating for small, frequent meals, etc., to mitigate the frequency of their reflux occurrence. Additionally, this study revealed that a 1-unit increase in BMI raises reflux risk by 36% (*OR* ​= ​1.36), which was similar to the findings of Nirwan JS et al.^31^ This association may stem from the prevalence of diaphragm uplift, increased abdominal pressure, etc., in most overweight patients, which increases the possibility of reflux occurrence. Therefore, effective measures should be taken to prevent and manage obesity. The present study also identified a 1-unit increase in Working Pressure raises reflux risk by 169% (*OR* ​= ​2.69), which is consistent with related studies;^32^ and the underlying reason for the analysis is that excessive mental stress may lead to gastrointestinal dysfunction, which is prone to overproduction of gastric acid for reflux. Furthermore, the results of the study also showed that the presence of patients with underlying disease was associated with the risk of reflux, consistent with the conclusions drawn by Dongfei Liang.^33^ This could be explained by the fact that gastro-esophageal reflux acts as a series of diversified syndromes that often coexist and interact with a variety of diseases, necessitating multifaceted preventive measures and heighten awareness among patients and their family members. In addition, this study showed that the risk of reflux increased when dysphagia and pain symptoms occurred in postoperative patients with esophageal cancer, a 1-unit increase in dysphagia raises reflux risk by 97% (*OR* ​= ​1.97), a 1-unit increase in pain raises reflux risk by 134% (*OR* ​= ​2.34), which is consistent with related studies.^34^ Possible reasons include residual esophageal motility dysfunction following esophageal cancer surgery, which can lead to dysphagia, and concurrently predisposes patients to reflux. Furthermore, whereas surgical trauma and the release of inflammatory factors cause pain and sleep disorders in patients,^35^ postoperative pain can lead to patients being in a highly stressful state over a long period of time, further increasing the risk of reflux occurrences. In this study, insomnia was negatively correlated with reflux symptoms, a 1-unit increase in insomnia decreases reflux risk by 46% (*OR* ​= ​0.54), The analysis revealed that insomnia patients experience frequent nighttime awakenings, leading them to adopt a supine sleeping position. Additionally, insomnia patients frequently rely on sedatives (such as benzodiazepines), whose muscle-relaxing effects can potentially diminish the temporary relaxation of the lower esophageal sphincter, thereby decreasing sensitivity to reflux symptoms and mitigating the risk of reflux occurrence. Regarding the multivariate logistic regression analysis, patients with comorbidities were negatively associated with reflux, a 1-unit increase in Presence of underlying disease decreases reflux risk by 57% (*OR* ​= ​0.43), which may be attributed to the following factors: (1) This study employed the GERDQ self-report questionnaire as the diagnostic criterion for reflux, and patients with comorbidities may have obscured or overlooked reflux symptoms due to other symptoms (such as chest pain or dyspnea). (2) Gastric acid secretion inhibition: Certain underlying conditions (e.g., chronic kidney disease, autoimmune diseases) are associated with gastric mucosal atrophy or the use of medications (e.g., proton pump inhibitors), which can reduce gastric acid secretion and lower the incidence of reflux; autonomic nervous system dysfunction: Diseases such as diabetes can lead to autonomic neuropathy, potentially weakening LES relaxation capacity and reducing reflux frequency. (3) Compensatory protective behaviors: Patients with underlying conditions may strictly adhere to reflux prevention measures (such as avoiding lying flat after meals or elevating the head of the bed at night) due to heightened health awareness, thereby offsetting the risks associated with their underlying conditions.

Studies have shown that^7^ postoperative symptom clusters in patients with esophageal cancer exhibit a certain degree of correlation, with multiple symptoms exhibiting synergistic effects and interrelationships, exacerbating pain and remarkably impairing quality of life. Consequently, it is imperative for healthcare providers to promptly assess patients, monitor and prevent the occurrence of related symptoms, foster active communication with patients and their families, and assist patients in effectively managing symptoms to promote disease recovery and enhance overall quality of life.

### Clinical utility of the predictive model

In this study, the ROC curve and calibration curve indicate that the model shows acceptable diagnostic performance. The factors in the model can be readily obtained from patient medical records or self-reports, rendering its seamless utilization in clinical settings. Based on these predictive factors, nursing staff can assess the risk of reflux in patients with esophageal cancer and tailored personalized nursing interventions accordingly. This approach provides essential guidance for clinical decision-making, facilitating optimal resource allocation, and potentially enhancing patient prognosis and quality of life. However, it is important to note that due to the heterogeneity of patient characteristics, hospital types, and treatment regimens, this model requires external validation before being applied to other populations so as to ensure its reproducibility. Moving forward, the feasibility and efficacy of the reflux warning model will be further determined through prospective cohort studies. Large-scale prospective multicenter studies will be conducted using stratified random sampling and dynamic monitoring techniques to further validate the model. Furthermore, while the model demonstrates high sensitivity (0.923), specificity in the validation group is suboptimal (0.568), indicating a relatively high false-positive rate. In clinical practice, it is recommended to implement optimized intervention strategies in clinical practice to avoid over-intervention. Risk stratification management should be adopted, with immediate 24-h pH monitoring to confirm reflux for high-risk patients (model probability ≥ 0.35) and initiate intensive interventions such as proton pump inhibitors (PPIs), positional requirements, and dietary control. For patients with moderate to low risk (probability < 0.35), short-term empirical PPI therapy should be administered, accompanied by guidance for patients to promptly perform self-monitoring of reflux, while providing basic health education and regular follow-up to reduce false-positive interventions and save medical costs.

### Limitations

This study has several limitations. First, it was conducted at a single center (Tianjin Medical University Cancer Institute and Hospital), and patients with severe postoperative complications were excluded. This may introduce selection bias and limit the generalizability of the findings to broader populations, particularly those with poorer baseline health or multiple comorbidities. Consequently, the model may underestimate the risk of reflux in high-risk groups. Second, variation in patients' adherence to postoperative follow-up may have introduced bias. Moreover, symptom assessment relied primarily on self-reported data obtained through the GerdQ questionnaire and retrospective medical records. The lack of objective monitoring (e.g., pH-impedance or endoscopy) raises the possibility of information bias due to recall or reporting inaccuracies. Logistic regression was selected as the modeling method based on its interpretability, clinical applicability, and appropriateness for the sample size and variable structure (398 patients and 29 clinically informed predictors). The model's transparency and statistical assumptions enabled rigorous evaluation, including collinearity diagnostics and the Hosmer–Lemeshow goodness-of-fit test. These characteristics align with the methodological rigor expected in clinical research. SHAP (SHapley Additive Explanations), although widely used in machine learning interpretation, was not employed due to its limitations in this context. SHAP assumes feature independence, which may not hold in clinical datasets; it does not support causal inference; and its computational demands may reduce interpretive reliability in smaller datasets. Finally, the retrospective design and moderate sample size limit the ability to assess the model's prospective predictive utility. The absence of real-time validation prevents assessment of its feasibility in routine clinical practice. Future studies should include prospective, multicenter cohorts, incorporate patients from diverse clinical settings, and employ dynamic monitoring to externally validate and optimize the model's clinical utility.

## Conclusions

In summary, age, body mass index, occupational stress, presence of comorbidities, dysphagia, insomnia, and pain were identified as independent risk factors for postoperative reflux in patients with esophageal cancer. An early warning model incorporating these factors demonstrated satisfactory predictive performance; however, external validation is necessary prior to clinical adoption. This model has potential utility for healthcare professionals to promptly stratify reflux risk following esophagectomy and to implement timely preventative interventions, thereby reducing reflux incidence and enhancing patient quality of life.

## CRediT authorship contribution statement

**Xueying Sun**: Conceptualization, Methodology, Data curation, Formal analysis, Writing. **Lirong Wei**: Conceptualization; Methodology; Data curation; Formal analysis; Original draft. **Huixia Li**: Supervision; Methodology; Review & editing. Qian Bai, Na Cao, Sihan Liu: Collect and verify data; Review & edit. **Yuqing Zhao**: Quality control of data; Statistical analysis. All participants provided written informed consent.

## Ethics statement

Prior to each follow-up visit, all 398 participants were informed of the study's purpose and significance. Written informed consent was obtained from each participant through the signing of a paper consent form. This study did not involve animal subjects or interventional human clinical trials and was therefore exempt from additional ethics committee approval. In accordance with the principles set forth in the Declaration of Helsinki, all participants voluntarily provided informed consent prior to enrollment. Participant anonymity and confidentiality were strictly maintained throughout the study. This research constitutes an extension of ethical review and has been approved by the Ethics Committee of Tianjin Medical University Cancer Hospital (IRB No. bc20250560).

## Data availability statement

The data that support the findings of this study are available on request from the corresponding author, HL.

## Declaration of generative AI and AI-assisted technologies in the writing process

No AI tools/services were used during the preparation of this work.

## Funding

This research was funded by the Tianjin Medical University Cancer Hospital Nursing Special Fund (Grant No. TJMUCH-H-2021-06) and the Tianjin Key Medical Discipline (Specialty) Construction Project (Grant No. TJYXZDXK-011A) in China. The funders had no involvement in the study design, data collection, analysis, interpretation, writing of the report, or the decision to submit the article for publication.

## Declaration of competing interest

The authors declare no conflict of interest.
